# Epizoötic Sore Mouth of Sheep in Montana

**Published:** 1895-07

**Authors:** R. N. Mead, Frederic Priest

**Affiliations:** Columbus, Ohio; Columbus, Ohio


### EPIZOÖTIC SORE MOUTH OF SHEEP IN MONTANA.

BY R. N. MEAD AND FREDERIC PRIEST,[1]
COLUMBUS, OHIO.

On October 12, 1894, Professor H. J. Detmers, of the Ohio State University, received a letter from Mr. F. Snyder, Musselshell, Montana, asking for information regarding a peculiar disease prevailing among the sheep, and especially among the lambs of his district. In the following we give the letter of

[1] Extract from Thesis at Department of Veterinary Science, Ohio State University.

Mr. Snyder and subsequent correspondence, together with a synopsis of our investigation and conclusions.

H. J. DETMERS, V. S.,
1315 Neill Avenue, Columbus, O.

DEAR SIR: I enclose a scab from the sore mouth of a lamb. The lambs in this State get the sore mouth about November; it lasts a month or six weeks, being fatal to many by reason of thinning them down so they will not stand the winter. Everyone takes them out of the band and feed hay for a month as the only remedy, as their lips swell so they cannot get their teeth to the grass.

It is contagious, if not infectious, for I have tried it two years, accidentally my pet lambs getting in with the sick ones just once or twice when they were nearly well in the one case, in the other when at the worst stage.

Feeding well lambs at the grain-trough after the others are gone, even after freezing weather, will sore the well ones' mouths. What is it? How do you prevent its appearance? Only lambs get it, sheep rarely. Grease makes it worse; swabbing the mouth three or four times thoroughly with one-quarter ounce of carbolic acid melted in a pound of grease helps them, but they fall away, and have to be kept in the hospital all winter in some cases. What do you think causes this? Respectfully yours,

F. SNYDER.

MUSSELSHELL, MONT., October, 1894.

We expressed our desire to investigate the disease, and through kindness Professor Detmers placed in our possession the above letter and scab. The letter was postmarked Musselshell, Mont., October 9, 1894. Received at Columbus, Ohio, October 12, 1 P.M., 1894.

The scab was about the size of a three-cent piece by one-eighth inch in thickness; the external surface gray to almost white, the internal surface dark brown. In the substance were many of the fine hairs of the lips, in an erect position, extending through and above the surface. On the surface there were also many little fissures, some of which were in depth equal to half the thickness of the scab, these radiating in an undulating manner from centre to circumference.

SHEEP NO. 1.—*October* 18, 1894, we procured a grade merino ewe, four years of age, weighing ninety-five pounds. A physical examination satisfied us that the animal was in good condition to enter the experiment as subject.

19*th*, A.M. Temp. 102.2° F. Following the taking of temperature came the inoculation, which consisted in scarifying the skin at the apex of the lower lip, at the corner of the mouth on the left side, also opposite the centre of external masseter muscle, and a point on inner surface of the hind leg, smearing over each of these points with some of the initial scab, which had been previously softened in water.

*20th*, A.M. Temp, 102.1° F. P.M. Temp. 102.3° F.

*21st*, A.M. Temp. 102.8° F. P.M. Temp. 102.8° F. The points of inoculation were healed over, and no visible signs of an infection were present. This led us to think that we had as a subject a non-susceptible animal. So our taking of temperatures and watching of the animal ceased.

*31st.* When feeding the animal, noticed a dark spot at the apex of the lower lip, and examination revealed a reddish-brown papule about the size of a grain of buckwheat; also two papules at the commissure inoculation, one on upper and one on lower lip. The cheek and leg inoculations were covered with brown scab, no papules.

*November 1st*, A.M. Temp. 102.6° F. P.M. Temp. 103.2° F. Animal has slight diarrhœa; apex papule much larger; commissure papules becoming ulcerous.

*2d*, A.M. Temp. 104.2° F. P.M. Temp. 103.8° F. Papules rapidly forming and becoming confluent; animal refuses food and water.

*3d*, A.M. Temp. 102.8° F. P.M. Temp. 104.6° F. Animal stupid; lymphatic glands swollen; papules nearly all ulcerous, and scabs being produced.

*4th*, A.M. Temp. 105.1° F. P.M. Temp. 104.9° F. Papules all advanced to the ulcerous stage, have become more or less confluent, and scabs being produced; no new papules forming.

*5th*, A.M. Temp. 104.2° F. P.M. Temp. not taken.

*6th*, A.M. Temp. 104.6° F. P.M. Temp, 104.8° F.

*7th*, A.M. Temp. 103.2° F. P.M. Temp. 102.8° F. Scabs getting thick, and the characteristic fissures appearing.

*8th*, A.M. Temp. 102.6° F. P.M. Temp. 102.1° F. Animal improving; eats well; swelling of glands disappeared; a rapid granulation beneath the scabs.

*9th*, A.M. Temp. 101.9° F.

*10th*, A.M. Temp. 101.6° F. Removed all scabs and placed in tubes as material for future inoculations.

*13th.* All causes have ceased to act, and a perfect recovery present. There are no scars to indicate any loss of substance. During the entire existence of the disease no trace of the morbid process extended into the mucous membrane or to the tougher skin, but remained localized to the soft skin of the muzzle.

14*th*. Placed sheep No. 1 in a clean pen in a new building 200 feet distant from the one in which it had been confined from the time of purchase until this 14th day of November. Put in the same pen with sheep No. 1 sheep No. 2, a grade merino ewe, four years old. Made no inoculation to sheep No. 2; this experiment will be conducted with a view to a natural infection.

*November* 15*th*, A.M. Temp. 102.8° F. P.M. Temp. 102.3° F.

16*th*, A.M. Temp. 102.2° F. P.M. Temp. 102.6° F.

17*th*, A.M. Temp. 102.2° F. P.M. Temp. 102.2° F.

18*th*, A.M. Temp. 102.3° F. P.M. Temp. 102.2° F.

19*th*, A.M. Temp. 102.2° F. P.M. Temp. 102.3° F. Five days after the sheep were placed in the pen together a papule about the size of a millet seed appeared on the right lower lip about one inch in front of the commissure of the mouth.

20*th*, A.M. Temp. 102.2° F. Papule increasing in size; second papule coming on left side about same point. P.M. Temp. 102.3° F.

21*st*, A.M. Temp. 102.2° F. P.M. Temp. 102.3° F.

22*d*, A.M. Temp. 102.3° F. P.M. Temp. 102.3° F.

23*d*, A.M. Temp. 102.3° F. P.M. Temp. 102.2° F. The first papules have become ulcerous, and the whole muzzle covered with small papules.

24*th*, A.M. Temp. 102.2° F. P.M. Temp. 102.3° F.

25*th*, A.M. Temp. 102.2° F. P.M. Temp. 102.3° F.

26*th*, A.M. Temp. 102° F. P.M. Temp. 102.1° F. All but two or three of the papules have advanced to the ulcerous stage, some covered with the thick characteristic scab.

27*th*, A.M. Temp. 102.3° F. P.M. Temp. 102.2° F.

28*th*, A.M. Temp. 102.6° F. P.M. Temp. 102.1° F.

29*th*, A.M. Temp. 102.4° F. P.M. Temp. 102° F. Removed scabs, rapid granulation beneath.

20*th*, A.M. Temp. 102.2° F. P.M. Temp. 102.1° F.

*December* 3*d*. All ulcers have healed, no scars remaining. It will be noticed that the temperature did not reach any abnormal height, nor did it vary greatly. The animal ate with comparatively little inconvenience, and no symptoms of disease were observed other than the sore mouth, which was more extensive than on sheep No. 1. Lymphatic glands not swollen, no diarrhœa, the morbid process extended neither into the mucous membrane nor to the tougher skin, but remained localized to the soft skin of the muzzle.

SHEEP NO. 3.—Sheep No. 3 is a yearling merino wether. *November* 15*th*, inoculated No. 3 at two points on the lower lip, the inoculating material being produced by inoculating nutrient gelatin in tubes with scab from Montana. The fourth culture remote from the scab was used for the inoculation. The culture was a mixed one, containing one short bacillus with rounded ends, one large and one small coccus, and one fungus.

*November* 15*th*, P.M. Temp. 103.4 F.°

16*th*, A.M. Temp. 103.6° F. P.M. Temp. 104° F.

17*th*, A.M. Temp. 103.6° F. P.M. Temp. 103.3° F.

18*th*, A.M. Temp. 103.3° F. P.M. Temp. 103.4° F.

19*th*, A.M. Temp. 103.3° F. P.M. Temp. 103.4° F.

20*th*, A.M. Temp. 103.2° F. Five days after inoculation small papule on lower lip at point of inoculation.

20*th*, P.M. Temp. 103.3° F.

21*st*, A.M. Temp. 103.2° F. P.M. Temp. 103.3° F.

22*d*, A.M. Temp. 102.5° F. P.M. Temp. 103.3° F.

23*d*, A.M. Temp. 103.4° F. P.M. Temp. 103.2° F.

24*th*, A.M. Temp. 103.2° F. P.M. Temp. 102.4° F. Papule has disappeared. It did not come to ulceration; however, it looked exactly like those on sheep 1 and 2.

25*th*, A.M. Temp. 102.5° F. P.M. Temp. 102.6° F.

26*th*, A.M. Temp. 102.3.° F. P.M. Temp. 103° F. Inoculated two points on lower lip with mixed cultures of micro-organisms which had been cultivated on potato.

*December* 10*th* (fourteen days after second inculation of sheep No. 3). Nothing developed.

22*d*. Placed sheep No. 3 in the pen with Nos. 1 and 2 with a view to natural infection.

*January* 6*th*. As yet no signs of a natural infection. Thinking that the inoculation with the remote culture that produced the papule had produced immunity, we now tried to solve this question by inoculating sheep No. 3 with scab from sheep No. 1.

12*th*. Six days after inoculation with scab, papule appeared at the point of inoculation.

13*th*. One papule on upper and two on lower lip.

14*th*. Many papules present.

15*th*. Older papules becoming ulcerous, and lips swollen considerably.

20*th*. The morbid process has reached the heighth of development, the ulcers covered with the thick, characteristic scab.

*31st.* Sheep No. 3 fully recovered, the temperature did not vary over a degree either way, no diarrhœa, no swelling of glands; was for a time a little indifferent and slow about eating hay. The morbid process extended neither into the mucous membrane nor into the tougher skin, but remained localized to the soft skin of the muzzle.

CONCLUSION. Although the papule produced by the culture inoculation did not course in the characteristic manner, it seems to have some bearing, as the animal was submitted to conditions favorable for a natural infection for a period of fifteen days without being infected. But if the animal was immune it was only to a slight degree, as a direct inoculation produced the disease in its characteristic form, after the usual period of incubation, five to six days.

We continued our experiments for the purpose of finding, if possible, the true cause of the disease, which we had reason to believe to be a specific micro-organism. Sheep being rather expensive, we tried to find some other susceptible animal less expensive to experiment with.

*March 12th.* We inoculated one young rabbit on the lower lip. Material used for inoculation was scab from sheep No. 1.

*18th* (six days after inoculation). Two small papules appeared at point of inoculation. From day to day the morbid process progressed from papule formation to ulceration, later to production of characteristic scab and heating. However, none but the two papules appeared.

*17th.* Inoculated rabbit No. 2 (an old male) with scab from sheep No. 2.

*23d* (six days after inoculation). Papule appeared, the morbid process progressed from day to day, spread to the upper lip, and the whole muzzle was covered with the characteristic scabs. The rabbit ate well, and showed no signs of being ill other than the sore mouth.

*April 9th.* Inoculated two young rabbits ([illegible] and 4) with scab from rabbit No. 1.

*15th* (six days after inoculation). Several small papules on upper and lower lips of rabbits 3 and 4.

With the above rabbits experimented with no signs of illness prevailed other than the sore mouth; nor did the morbid process extend into the mucous membrane or tougher skin, in any case.

We inoculated nutrient gelatin in tubes with scab from Montana, and in twenty-four to forty-eight hours had a strong germ development, many varieties being present. The impure cultures grow more rapidly at surface of gelatin, but grow all along the puncture. They begin to liquefy the gelatin at top and proceed downward in funnel shape, the liquefied gelatin being turbid. In course of two or three weeks the whole mass of gelatin is liquefied, at the bottom of which lies a yellowish, sediment-like mass, which is composed of bacteria and large and small cocci. From such cultures we made plate cultures, the fourth plate being necessary to separate them.

In twenty-four to forty-eight hours we find two distinct growths, both superficial colonies which grow beneath the surface of the gelatin. One of the colonies grew very rapidly, at first white, later having a blush halo about it. This one liquefies the gelatin rapidly. This is a pure colony of bacteria, short, thick, straight rods with rounded ends. The other does not grow so rapidly, and does not liquefy the gelatin. The colonies first appear as little dots, as they advance they show a yellowish coloration; these are pure colonies of medium-sized cocci, which appear singly or in twos.

Tube cultures from Montana scab, also from sheep Nos. 1, 2, and 3, and rabbits, all have the same characteristic growths on the gelatin plate, the same two colonies (bacilli and cocci) develop in the characteristic manner.

We have inoculated rabbits on the lip with the mixed cultures which were produced by inoculating gelatin tubes with scab, and in each case this inoculation failed to have any perceptible effect. Inoculated rabbits with each of the pure colonies (bacteria and cocci) grown on the gelatin plates; these failed to produce the disease.

From the above experiments and their results we have arrived at the conclusion that the micro-organisms which we have grown on gelatin are not the primary cause of the disease in question, although they undoubtedly play an important part in the morbid process.

We directed a letter to Mr. Snyder, of Montana, asking his help in that he give us an idea of the conditions prevailing at times of outbreak of the disease in question (mouth disease of sheep). November 20 came the following reply:

DEAR SIRS: Yours of the 11th came duly. I will help you all I can, but cannot promise prompt answers, as I am very busy and twelve miles from the office.

This disease is prevalent all over the Musselshell Valley to my knowledge, and I think all over the State; looked upon as one of the things lambs must have, and not much said about it. Sheep are herded in bands varying from 1000 to 4000, and this sore mouth comes mostly to lambs that have not been weaned, but allowed to let the ewes wean them or not, as they please.

The time varies because there is no set time to put sheep in their winter range, which is a dry camp, *i. e.*, where they must eat snow or, that failing, must be driven several miles every third day to water. It has been attributed to salting them, thus freezing their mouths; but lambs raised at the house, that get salt every day, never get the disease unless brought into contact with the lambs from the bunch which have sore mouth; even then they must eat with the diseased ones; if separated by a fence they are safe. But sooner or later, even when they are four years old, they are liable to take it when exposed to it; but they never have it twice.

The use of those applications of carbolic tallow, one-half ounce of carbolic acid to a pound of tallow, swabbed on their mouths three times, then take them off the range and feed hay uutil swelling goes out of their lips so they can pick the grass again; feeding them oats and hay during that period, usually a month.

Fat lambs have few sores, their lips do not swell badly, and it lasts but two weeks. Some that are poorer than average will have it six weeks, and some die because they cannot eat hay and oats. If you do nothing for them, leave them on the range, there may be a 50 per cent. loss if the weather turns bad, as they chill and die.

Our lambs this year are not in their winter range, will not be there before the 1st of January, as so far we have no snow. So they may not have it at all. As they are about weaned they will be turned back to the ewes on the 26th. They are now drifting out of sight, so I will have to close and attend to my herding. Respectfully,

F. SNYDER.

GENTLEMEN: Your letter of February 18th received in due time, and I am very sorry I could not give it more prompt attention, although I have little or nothing to write that can be of use to you.

"Sore mouth" is the only name I have heard for the disease you describe, although I do not know that it ever extends to the mouth proper. My opportunity for observing it has been very limited, as we have had it among our sheep only twice, and each time to a very limited extent, affecting, so far as noticed, only a few that were in an unthrifty condition. Each time, too, it disappeared without treatment, and without greater loss than might have occurred in its absence, the sheep only being changed from feeding on the range to hay and water in the yards, with sometimes a little grain. Late fall or early winter is the season when the disease makes its appearance, and at that time we always have some unthrifty sheep to pick out of the main flocks and feed in the yards, as above mentioned. You are doubtless aware that the most of our sheep graze on the open plains winter as well as summer. It is only weak and unthrifty sheep that are kept and fed in the yards, and these are termed "invalids" or "hospital sheep." With regard to cause or prevention I know nothing more than may be suggested by the above allusions to the conditions of the sheep which were, in our experience, affected.

The Hon. E. Beach, Helena, Mont., has had a more extended experience with the disease, and has been compelled to adopt treatment for it, which, I believe, has consisted of applications of a carbolic salve. It could do no harm to address him in regard to it, and it might elicit information of much value to you. Regretting that I am unable to contribute more extensive and more precise information, I remain, Yours very respectfully,

C. Y. LACY.

LACY, CHOTEAU CO., MONT., March 14, 1895.

It is a very difficult task, in fact beyond our limit, to give a perfect and intelligent description of all the conditions prevailing where this disease has its home; nor can we state what localities are affected or exempt. Can only refer to above letters.

A lack of time and means has prevented a thorough investigation conducted in a proper manner. So we will be compelled to draw our conclusions from our narrow experiments and the embodied letters. We are convinced that the disease is strictly a contagious one, and limited to a certain part, *e. g.*, the softer skin about the muzzle. The disease is characterized by the rising of papules, advances to ulceration, then incrustation, and, finally, in the majority of cases, to a rapid recovery. A fatal termination will be a small per cent. if properly cared for.

TREATMENT.—A medicinal treatment will be of little or no value in most cases, as the disease will run its course in from two to three weeks without a medicinal treatment, provided they are protected from severe and stormy weather, and get proper food. Applications of tincture of iodine, carbolic acid, creolin, etc., must be repeated for several times to benefit the animals materially. In large flocks this would require much labor and expense, where the disease is permitted to run through the flock by natural infection, as the disease would remain in the flock for several months. Thus flock-owners would suffer losses due chiefly to severe winter weather. We would suggest to take the lambs up at convenient time before the severe weather sets in and inoculate them with the disease-producing material, which may be collected from sheep affected in a mild form, then feed hay and grain, with clean water for drinking. Following this procedure the disease will be disposed of in about twenty-one days; the period of incubation being five to six days, reaches its height in about ten days more, a recovery takes place in five to six days (according to our experiments). If any severe cases occur it would be advisable to apply some of the medicinal agents before mentioned.

